# Adherence to and Motivations for Complying With Public Health Measures Among Adolescents During the Coronavirus Disease (COVID-19) Pandemic in Canada

**DOI:** 10.1177/00332941231201355

**Published:** 2023-09-21

**Authors:** Stephanie G. Craig, Christina L. Robillard, Megan E. Ames, Samantha Feldman, Debra J. Pepler

**Affiliations:** Department of Psychology, 3653 University of Guelph, Guelph, ON, Canada; Department of Psychology, 8205 University of Victoria, Victoria, BC, Canada; Department of Psychology, 7991 York University, Toronto, ON, Canada

**Keywords:** COVID-19, mask wearing, physical distancing, social interaction, youth

## Abstract

**Background:** Public health measures (e.g., minimizing social interactions, social distancing, and mask wearing) have been implemented in Canada to reduce the transmission of COVID-19. Given that adolescents may be a high-risk demographic for spreading COVID-19, this study investigated adherence to and motivations for complying with public health measures among Canadian youth at two points of the COVID-19 pandemic. **Methods:** Adolescents (*N* = 1,484, 53% girls, *M*_age_ = 15.73 [*SD* = 1.41]) completed an online survey in either Summer 2020 (Cohort 1 [C1]; *n* = 809, 56% girls) or Winter 2020/2021 (Cohort 2 [C2]; *n* = 675, 50% girls). We investigated differences in adherence across cohorts using independent sample *t*-tests and predictors of adherence using a path analysis. **Results:** Youth engaged in similar levels of social interaction in C1 and C2. Relative to adolescents in C1, adolescents in C2 reported more mask wearing, but less social distancing. Social responsibility was associated with adherence to almost all public health measures across both cohorts, with one exception: it did not predict minimizing social interactions in C2. Not wanting to get sick predicted minimizing social interactions and mask wearing. Concern with population health predicted adherence to all public health measures in C1 and all but mask wearing in C2. Maintaining social ties was negatively associated with minimizing social interactions in both cohorts, and with social distancing in C1. **Conclusions:** Youth engaged in more mask wearing but less social distancing as the pandemic progressed. Social responsibility and not wanting to get sick were consistent predictors of adherence to most public health measures throughout the pandemic. Youth shifted away from adhering to mask wearing measures due to concern with population health over the course of the pandemic. These results can inform targeted campaigns to bolster compliance with public health measures among adolescents.

## Introduction

The coronavirus disease 2019 (COVID-19) was declared a global pandemic by the World Health Organization (WHO) on March 12, 2020. Since then, seriously ill individuals requiring intensive care have put considerable stress on the healthcare system (McMahon et al., 2020; Wilbiks et al., 2021). Public health measures (e.g., minimizing social interactions, social distancing, and mask wearing) have been implemented to mitigate the burden of COVID-19 on the healthcare system by reducing infection rates and associated deaths. However, it remains unclear whether adherence to and motivation for complying with these public health measures changed as the COVID-19 pandemic progressed. It is essential to address this knowledge gap among adolescents in particular, as they have been identified as a potentially high-risk demographic for nonadherence to public health guidelines and spreading the virus, both in research (Bhatt et al., 2022; Jaureguizar et al., 2021) and the media (Kelly, 2020). Thus, the current study investigated adolescents’ adherence to and motivations for complying with public health measures at two different points of the COVID-19 pandemic (Summer 2020 and Winter 2020/2021) in Canada.

### Public Health Measures and Adolescents

The media has generally painted an unfavorable picture of adolescents disobeying public health policies during the COVID-19 pandemic (e.g., holding parties; Kelly, 2020). Some research has supported this notion, showing that youth are less compliant with public health measures than adults (Jaureguizar et al., 2021). Consistent with developmental theory (Elkind, 1967), adolescents may be less likely to engage in public health policies than adults due to lower risk perception (e.g., believing they are unlikely to contract COVID-19 or develop severe symptoms; Alberts et al., 2007). Furthermore, as friendships are increasingly important during adolescence (Waldrip et al., 2008), it is possible they face increased peer pressure to buck public health requirements. Further complicating this matter, youth tend to be more impulsive than adults (Romer, 2010), which may result in preference to socialize with friends rather than keep their family and loved ones safe by adhering to public health guidelines.

Contrasting this, the health belief model can help us to understand why adolescents may adhere to public health measures. This model has been used since the 1950s to understand adherence to public health measures (Becker, 1974). According to this model, there are two elements to health-related behavior: the desire to avoid illness and the belief that specific public health actions will prevent or cure illness. This model proposes that the probability of a person adhering to recommended public health behaviors depends on several key factors, such as an individual’s perceived threat to the illness (i.e., perceived susceptibility), the belief of consequences (i.e., perceived severity), potential positive benefits of action (i.e., perceived benefits), potential negative outcomes of the behavior (i.e., perceived barriers to actions), guidance people receive (i.e., cues to action), and the confidence in the ability to succeed (i.e., self-efficacy).

Limited research has systematically investigated adolescents’ adherence to and motivations for complying with public health measures during the COVID-19 pandemic. Inconsistent with media portrayals of youth, Oosterhoff et al. (2020) documented high rates of social distancing among American youth early in the pandemic (i.e., April 2020). Specifically, 83.5% of adolescents reported engaging in social distancing “a lot” or “a great deal” (Oosterhoff et al., 2020). Another study from the same period found that 95.0% of youth reported engaging in social distancing; however, 62% reported breaking the guidelines at least once (Dunn et al., 2021). In terms of mask wearing, one study from Ireland found that 92% of adolescents reported wearing a mask “most of the time” or “always” during the pandemic (Nearchou et al., 2022). In a Canadian sample assessed during the 2020/2021 school year, Patte and colleagues (2022) found that youth generally supported mask mandates, with 85.6% of youth acknowledging that masks prevent transmission of COVID-19 and 64.5% supporting mask requirements in schools.

There are a wide range of reasons that youth engaged in social during the COVID-19 pandemic, including intrinsic (i.e., social responsibility) and extrinsic (i.e., governmental sanctions and parental enforcement) motivations (Oosterhoff et al., 2020). In addition, research by Oosterhoff and colleagues (2020) found that youth who endorsed these motivations engaged in more social distancing than those who did not endorse these motives. However, some other intrinsic motivations, such as wanting to avoid personal sickness and getting others sick, were not associated with social distancing (Oosterhoff et al., 2020). These findings suggest that adolescents may be more strongly motivated to adhere to social distancing measures for social and societal reasons than health-related reasons. When examining motivations for mask wearing, however, some studies have found support for health-related motivations. For example, Patte and colleagues (2022) found increased odds of supporting mask use in indoor public spaces if the youth reported concerns about their own and family’s health, as well as perceived COVID-19 to be a risk and had discussions with others regarding ways to prevent infection. Another study that examined adolescents’ intention to comply with public health measures (e.g., mask wearing, social distancing, minimizing social interactions, frequent handwashing, etc.) found that increased compliance to these measures was associated with knowing more about protective measures, perceiving COVID-19 to be more detrimental to their own health, and believing the measures worked (Reinhardt et al., 2022). To our knowledge, no research has examined adherence to and motivations for complying with minimizing social interactions, social distancing, and mask wearing independently.

### Public Health Measures Throughout the Pandemic

Adherence to and motivation for complying with public health measures may have shifted throughout the pandemic, partly due to mixed messaging from public health organizations. When COVID-19 initially spread to North America in February 2020, the WHO stated that only people with symptoms of COVID-19 and those caring for them should wear masks (Lewis, 2022). The scientific community was concerned that mask wearing would create a false sense of safety and reduce adherence to social distancing measures (Tso & Cowling, 2020). Furthermore, the medical community was concerned that mask wearing could exacerbate COVID-19 transmission if not worn or disposed of properly (Lewis, 2022; Tso & Cowling, 2020). The WHO changed their stance on masks several times throughout 2020 (e.g., World Health Organization, 2020), and only within the last months of 2021 did they acknowledge the airborne nature of COVID-19 and recommend mask wearing for both symptomatic and non-symptomatic individuals (Lewis, 2022). This messaging may have contributed to relatively lower levels of adherence to and motivation for mask wearing early in the pandemic, relative to later in the pandemic when masks were strongly recommended or mandated. At the same time, pandemic fatigue and social isolation may have increased among adolescents as the pandemic progressed, which could have reduced adherence to public health guidelines. Consistent with these ideas, some research found that rates of compliance changed over time. One longitudinal study from the United Kingdom found that while adolescents were quite compliant with social distancing measures early in the pandemic, adherence waned by the second lockdown during early 2021 (Aksoy, 2022). Likewise, Franzen and Wöhner (2021) found that compliance for social distancing decreased from 94.5% to 87.6% from the first to second wave of the pandemic in Switzerland. However, a lack of longitudinal or cohort comparisons for mask wearing make it difficult to assess whether increased requirements for masks was associated with compliance (e.g., Larsen et al., 2021). It is important to empirically investigate this possibility, as this knowledge could help public health agencies identify ways of bolstering public health compliance among adolescents both now and in future outbreaks or pandemics.

### The Present Study

To date, no known research has investigated both adherence to and motivations for complying with COVID-19 public health measures among adolescents living in Canada. Moreover, limited research has investigated how this may have changed as the COVID-19 pandemic progressed. Addressing this knowledge gap could inform public policy and potentially improve targeted campaigns. Our study aimed to address this gap using two cohorts of youth – one assessed early (i.e., Summer 2020) and one assessed later (i.e., Winter 2020/21) in the pandemic. We had the following research questions: (1) to what extent did youth adhere to public health guidelines and what were their motivations; (2) did adherence to and motivations for complying with public health measures differ across cohorts (i.e., for youth early in the pandemic versus later in the pandemic); and (3) what motivations predicted adherence to public health measures across cohorts? Canada experienced its first and second wave of the pandemic at these time points, and it was then that social distancing and mask wearing, respectively, were first mandated.

## Method

### Participants and Procedure

All study protocols were approved by the authors’ Institutional Research Boards. Participants were drawn from 1484 adolescent living in Canada (53% girls, 6% *trans+*, *M*_age_ = 15.73 [*SD* = 1.41], 72% White) who were either recruited for Cohort 1 (C1) in Summer 2020 (i.e., June 17 to July 31, 2020) or Cohort 2 (C2) in Winter 2020/2021 (i.e., November 15, 2020, to February 28, 2021). C1 participants were 809 adolescents (56% girls, 5% *trans+, M*_age_ = 15.67 [*SD* = 1.37], 74% White) and C2 participants were 675 adolescents (50% girls, 6% *trans+*, M_age_ = 15.80 [*SD* = 1.46], 70% White). Full demographic characteristics can be found in Table 1. Both samples were consistent with the Canadian population in terms of gender and racial/ethnic identity (Statistics Canada, 2017). Adolescents were recruited through social media platforms (e.g., Facebook, Twitter, Instagram, and Reddit), including social media posts by community organizations that partner in research with the primary investigator (e.g., Big Brothers Big Sisters Canada). Informed consent was obtained from the adolescents. To ensure competency, youth were required to answer two questions pertaining to the risks and benefits to the study (e.g., risk of becoming stressed in response to some of the questions; benefit of contributing to research or reflecting on their experiences) and two questions regarding the purpose of the outlined study (Friedman et al., 2016). Participants who completed the survey were entered into a draw for incentives (i.e., gift cards).

**Table 1.** Sample Demographic Variables.

Demographics/Variables	Cohort 1 (*n* = 809) *n (*%)/*M* (*SD*)	Cohort 2 (*n* = 675) *n* (%)/*M* (*SD*)
*Age*		
*Mean*	15.67 (1.37)	15.80 (1.46)
*Range*	12 to 18	12 to 18
*Gender*		
*Girls*	455 (56.2)	335 (49.7)
*Boys*	313 (38.7)	298 (44.1)
*Trans+*	41 (5.1)	42 (6.2)
*Ethnicity*		
*White*	598 (74.2)	474 (70.2)
*Asian (East and Southeast)*	71 (8.8)	87 (12.9)
*Indigenous/Metis/First Nations*	54 (6.7)	27 (4.0)
*Black*	16 (2.0)	35 (5.2)
*Latin*	12 (1.5)	8 (1.2)
*Other/Mixed Ethnicities*	55 (6.8)	44 (6.5
*Location*		
*Ontario*	274 (33.9)	276 (40.9)
*British Columbia*	117 (14.5)	101 (15.0)
*Alberta*	160 (19.8)	121 (17.9)
*Manitoba and Saskatchewan*^a^	90 (11.1)	60 (8.9)
*Quebec*	66 (8.1)	52 (7.7)
*Atlantic Provinces*^b^	98 (12.1)	62 (9.2)
*Yukon and Northwest Territories*^c^*	4 (.5)	3 (.4)
*Living situation*		
*At home with parent(s)*	770 (95.2)	624 (92.4)
*Foster/group/other*	39 (4.9)	51 (7.6)
*Family composition*		
*Two caregivers*	561 (69.7)	459 (68.3)
*One caregiver*	178 (22.1)	178 (26.5)
*Other*	66 (8.2)	35 (5.2)
*School attendance*		
*Online*	679 (83.8)	141 (22.0)
*Mixed (online and in-person)*	60 (7.4)	247 (38.5)
*In-person*	16 (2.0)	204 (31.8)
*None*	54 (6.7)	27 (4.0)

^a^Provinces combined due to low *n* and similar public health implementation.

^b^Atlantic Provinces include Nova Scotia, New Brunswick, Prince Edward Island, and Newfoundland and Labrador.

^c^There were no respondents from Nunavut.

### Measures

#### Adherence to Public Health Measures

Adherence to public health measures was measured with three items adapted from a social distancing scale used in previous research (Oosterhoff et al., 2020). To measure social interactions, participants were asked: “*How often in the past month did you socialize in person with someone outside your immediate household or allowable social bubble?*”. To measure social distancing, participants were asked: “*When you saw people outside your household, how often did you maintain 6 feet (2m) distance?*”. To measure mask wearing, participants were asked: “*In the past month, to what extent did you wear a mask in public? (defined as being outside of your home)?*”. Items were rated on a 5-point Likert scale ranging from zero (*Not at all*) to 4 (*A great deal*). The social interaction item was reverse coded, such that a higher score indicated fewer social interactions and therefore greater adherence to public health measures. There was an administration error in C1 for the mask wearing item, such that participants were not given the option to select “A great deal.” To increase consistency between the mask wearing variable in C1 and C2, we collapsed the response categories of “A great deal” and “A lot” for this variable.

#### Motivations to Adhere to Public Health Measures

Participants were asked about their motivations for adhering to public health measures (adapted from Oosterhoff et al., 2020), as follows: “*Which of the following factors influence your decision to follow social distancing and public health guidelines (*e.g.*, wearing a mask, washing your hands, not touching your face) related to COVID-19? (select all that apply)*”, with responses coded as zero (*No*) and 1 (*Yes*). Youth rated nine factors, such as “*My province/city is in lockdown*”, “*It is socially responsible*”, and “*I don’t want to personally get sick.*” In addition, we included an “*Other*” option with an open-ended text box to allow participants to provide more information. Similar to previous research using this scale (Oosterhoff et al., 2020), we found that a small percentage of youth (8.9%) endorsed this item. A review of the responses indicated that most were duplicated (2.2%; e.g., “*Don’t want to get others sick*” duplicated “*It is socially responsible*”) or the COVID-19 stress scale (3.5%; see below; e.g., “*My relative is high risk*” duplicated “*My family member’s health*”). Thus, these responses were removed from the analysis to avoid redundant measurement. Other responses included adolescents indicating that they were not following public health measures because they did not believe COVID-19 was serious or felt that mask wearing infringed upon their rights, that they liked hiding behind a mask, or that they just wanted the pandemic to be over. Given the small number of these responses (i.e., *n* < 5), they were not analyzed further.

### COVID-19-Related Stress

COVID-19-related stress was measured using 12 items from the Statistics Canada COVID-19 Stress Scale (Findlay et al., 2020). Participants were asked how concerned they were about the impact of COVID-19 on various topics on a 4-point Likert scale ranging from zero (*Not at all*) to 3 (*Extremely*). The authors created two subscales (Craig et al., 2023): family health-related concerns (3 items; “*My own health,*” “*My family member’s health,*” “*Vulnerable people’s health [*i.e.*, grandparents]*”) and population health-related concerns (3 items; “*The country’s population health,*” “*World’s population health,*” “*Overloading the healthcare system*”). A mean score was calculated for both subscales, and the internal consistencies were .72 for family health-related stress and .84 for population health-related stress. Of the remaining six items on the COVID-19 stress scale, only one other item was examined; given that we were interested in whether stress around maintaining friendships is associated with adherence to public health measures (Waldrip et al., 2008), the item “*Maintaining social ties*” from the COVID-19-related stress measure was included.

### Data Analysis Strategy

Analyses were performed in SPSS Version 27 (IBM Corp, 2020) and Mplus Version 8.7 (Muthén & Muthén, 1998-2017). First, descriptive statistics were conducted to address Research Question (RQ) 1 examining the extent to which youth adhered to public health measures and what their motivations were in each cohort (i.e., C1 and C2). Second, chi-square (χ^2^) analyses and an independent sample *t* test were conducted to address RQ2 regarding whether there were differences in adherence to and motivations for complying with public health measures across C1 and C2. Finally, path analysis was employed to examine RQ3 regarding which motivations predicted adherence to public health measures in C1 and C2. More specifically, one unconstrained path analysis equation was constructed in Mplus, whereby adherence to each public health measure was simultaneously predicted by motivations, COVID-19-related stress, and covariates (i.e., age, girl gender, *trans+* gender). We also controlled for the number of weeks since the beginning of the study to account for the potential waning effect of time under public health measures. To examine whether the associations between motivations and COVID-19-related stress with adherence to public health measures differed across cohorts, we ran one constrained model where each parameter was set to be equal across cohorts and performed a Satorra-Bentler scaled χ^2^ difference test to assess if the model fit significantly worsened compared to the unconstrained model. We then released each parameter one at a time and tested whether the model fit was significantly different using the same Satorra-Bentler scaled χ^2^ difference test. If significant, the parameter was considered significantly different across cohorts. The final model included all significantly different pathways unconstrained (i.e., allowed to vary), whereas pathways that were not significantly different were constrained to be equal across the cohorts.

The unconstrained and constrained path analysis models were fit using full information maximum likelihood (FIML) to handle missing data and a robust estimator (MLR) to handle the non-normal distribution of the data. Goodness-of-fit indices included the chi-square, root mean square error of approximation (RMSEA), comparative fit index (CFI), and standardized root mean square residual (SRMR). Given that the significance level of the chi-square is dependent on sample size, we evaluated the model based on the CFI, RMSEA, and SRMR. A model with CFI values >.90 were considered to have acceptable fit and a model with a CFI >.95 were considered to have good fit; RMSEA values <.08 indicated acceptable fit and <.05 indicated good fit (Bentler & Bonett, 1980; Hu & Bentler, 1999). As we examined a high number of associations, only those with *p* < .01 were considered significant in the final model.

## Results

### RQ1: To What Extent Did Youth Adhere to Public Health Guidelines and What Were Their Motivations?

Rates and mean levels of adherence to public health guidelines in each cohort are presented in Table 2. Specifically, 49.1% (*n* = 389) of youth reported socializing “*Not at all*” or “*A little*” with individuals beyond their immediate household or allowable social bubble in C1, whereas 42.7% (*n* = 282) endorsed minimizing social interactions in C2. In terms of social distancing, 51.2% (*n* = 406) of youth said that they maintained a 6 feet (2 m) distance from those beyond their household “*A great deal*” or “*A lot*” in C1, whereas 43.0% (*n* = 284) endorsed one of these responses in C2. Furthermore, 40.2% (*n* = 318) of adolescents reported wearing a mask in public “*A great deal/a lot*” in C1, whereas 93.9% (*n* = 621) endorsed this response in C2.^1^ The proportion of youth who endorsed each motivation for adhering to public health measures, as well the mean endorsement of COVID-19-related stressors is reported in Table 3. The most common motivation for adhering to public health measures was social responsibility (C1: 69.7%, *n* = 553; C2: 80.8%, *n =* 534) and not wanting to get sick (C1; 51.6%, *n* = 409; C2: 59.5%, *n* = 394). Correlations between study variables in each cohort are presented in Supplemental Table 1.

**Table 2.** Adherence to Public Health Measures by Cohort.

	Cohort 1 (*M, SD/n*, %)	Cohort 2 (*M, SD/n*, %)
How often in the past month did you socialize in-person with someone outside your immediate household or allowable social bubble?		
Mean (*SD*)	2.19 (1.16)	2.11 (1.20)
A great deal	92 (11.6)	92 (13.9)
A lot	124 (15.6)	97 (14.7)
Somewhat	188 (23.7)	190 (28.7)
A little	317 (40.0)	211 (31.9)
Not at all	72 (9.1)	71 (10.7)
When you saw people outside of your household, how often did you maintain 6 feet (2 m) distance?		
Mean (*SD*)	2.44 (1.18)	2.19 (1.23)
Not at all	48 (6.1)	75 (11.4)
A little	135 (17.0)	117 (17.7)
Somewhat	204 (25.7)	184 (27.9)
A lot	229 (28.9)	176 (26.7)
A great deal	177 (22.3)	108 (16.4)
In the past month, to what extent did you wear a mask in public? (Defined as being outside of your home)?		
Mean (*SD*)	1.86 (1.13)	2.91 (.37)
Not at all	143 (18.1)	3 (.5)
A little	145 (18.3)	11 (1.7)
Somewhat	186 (23.5)	26 (3.9)
A lot/great deal	318 (40.2)	621 (93.9)

Note. Percentages are calculated based on the number of youth who answered each item.

**Table 3.** Endorsement of Motivations for Adhering to Public Health Measures by Cohort and Differences (X^2^) Between Cohorts.

	Cohort 1	Cohort 2	*X* ^*2*^
Motivations	(*n*, %)	(*n*, %)	
My province/city is on lock down	236 (29.8)	239 (36.2)	6.71**
My parents are making me	264 (33.3)	192 (29.0)	3.02
My friends told me I should	48 (6.1)	45 (6.8)	.34
It is socially responsible	553 (69.7)	534 (80.8)	23.33***
I don’t want to get sick personally	409 (51.6)	394 (59.6)	9.40***
There is nothing going on anyway	68 (8.6)	35 (5.3)	5.89
I Prefer to stay at home	276 (34.8)	186 (28.1)	7.39**
I don’t want to be socially judged	146 (18.4)	123 (18.6)	.01
Work/home responsibilities	156 (19.7)	135 (20.4)	.13
Missing	16 (2.05)	14 (2.1)	
COVID-19 stressors	(*M, SD*)	(*M, SD*)	
Family’s health	1.95 (.84)	2.02 (.82)	
Population health	1.55 (.81)	1.62 (.83)	
Maintaining social ties	1.42 (.97)	1.56 (.93)	

Note. Percentages are calculated based on the number of youth who answered each item. **p < .01, ***p < .001; all *X*^*2*^ df = 1.

### RQ2: Did Adherence to and Motivations for Complying with Public Health Measures Differ Across Cohorts?

The extent to which adolescents socialized with people beyond their immediate household or allowable social bubble did not significantly differ by cohort (*t* [1452] = 1.35, *p* = .176). That said, adolescents engaged in significantly more social distancing in C1 than C2 (*t* [1451] = −4.01, *p* < .001). An opposite pattern emerged for mask wearing, such that youth reported significantly more mask wearing in C2 than C1 (*t* [989.66] = 24.65, *p* < .001).

In terms of motivations, the χ^2^ analysis (see Table 3) revealed that compared to youth in C1, a higher proportion of adolescents in C2 endorsed the province being in lockdown, being socially responsible, and not wanting to get personally sick as reasons for adhering to public health measures. As it is important to consider living situation for the item “*My parents are making me*,” we conducted a post-hoc analysis examining whether this item differed based on a youth’s living situation (i.e., living at home with parents versus other living situations such as kinship or foster care). Those living at home were more likely to endorse their parents making them adhere to public health measures as a motivation (34% and 30% respectively across C1 and C2).

### RQ3: Which Motivations Predicted Adherence to Public Health Measures Across Cohorts?

All outcomes were examined in the same path analysis equation model, with the dependent errors correlated. The unconstrained model had good fit, χ2 [6] = 2.72*, p* = .843, RMSEA = .00 [90% CI .00, .027], CFI = 1.0, SRMR = .005. Likewise, the constrained model had acceptable fit, χ^2^ [54] = 148.66, *p* < .001, RMSEA = .05 [90% CI .04, .06], CFI = .85, SRMR = .06. Based on model fit and the use of dichotomous variables for the motivations measure, the correlations between the predictors in the model were set to zero. However, the Satorra-Bentler scaled χ^2^ difference test was significant (*TRd* = 144.11, Δ*df* = 48, *p* < .001), indicating that the unconstrained model was a significantly better fit of the data. This suggests that parameter estimates differed by cohort, and as such, the unconstrained model was interpreted. We then released one parameter at a time to check for significant differences using the Satorra-Bentler scaled χ^2^ difference test. The final model indicated 10 pathways to be unconstrained across the cohorts, as indicated in Table 4. The final model fit the data well χ^2^ [42] = 90.36, *p* < .001, RMSEA = .04 [90% CI .03, .05], CFI = .92, SRMR = .03.

**Table 4.** Results From Path Analysis Examining Predictors of Adherence to Public Health Measures by Cohort.

	Minimizing social interactions	Social distancing	Mask wearing
	*R*^2^ = .07/.04	*R*^2^ = .09/.06	*R*^2^ = .14/.13
	β	β	β
Demographics			
Age	.02	−.06	−.01
Girl gender	.07	−.03	.01
Trans+ gender	.05	.00	.12
Weeks	.00	.03	.11***/.01
Motivations			
Lockdown	.05	.01	.02
Parents are making me	.00	−.01	.09**/-.02
Friends said I should	−.01	.03	.00
Socially responsible	.12**/.06	.13***	.14***/.23***
Don’t want to get sick	.09**	.06	.11***/13***
Nothing else going on	.03	−.06	−.01
Prefer to stay home	.11**	.05	−.04**/-.10***
Socially judged	−.04	−.03	.00
Work responsibilities	−.07	.01	.00
COVID-19 stress			
Family health	−.02	.09***	.04
Population health	.09**	.13***	.16***/.07
Maintaining social ties	−.09**	−.10**/.03	.00

Note. where cell has no slash, parameter was not significantly different between Cohort 1 and 2; slashes indicate parameter that had significant difference between Cohort 1 and 2 based on Satorra-Bentler scaled χ^2^ test, numbers on left side of slash represent Cohort 1, number on right side of slash represent Cohort 2. Weeks, number of weeks since the beginning of the study; β, standardized coefficient.

**p < .01, ***p < .001.

#### Minimizing Social Interactions

In C1 and C2, not wanting to get sick, preferring to stay home, and being concerned with population health were positively associated with minimizing social interactions. Social responsibility was positively associated with minimizing social interactions in C1, but not in C2. Concern with maintaining social ties was negatively associated with minimizing social interactions in C1 and C2.

#### Social Distancing

In C1 and C2, social responsibility, concern with family health, and concern with population health were positively associated with social distancing. Concern with maintaining social ties was negatively associated with social distancing in C1, but not in C2.

#### Mask Wearing

In C1, social responsibility, not wanting to get sick, parental enforcement, and concern with population health were positively associated with mask wearing. In C2, social responsibility and not wanting to get sick also predicted mask wearing; however, parental enforcement and concern for population health did not. Preferring to stay home was negatively associated with mask wearing in both C1 and C2.^2^

## Discussion

We examined adherence to and motivations for complying with public health measures (i.e., minimizing social interactions, social distancing, and mask wearing) among two cohorts of adolescents assessed at different points of the COVID-19 pandemic (i.e., Summer 2020 and Winter 2020/2021) in Canada. The media has painted an unfavorable picture of adolescents disobeying public policies (Kelly, 2020), and some research has documented less compliance with COVID-19 public health policies among youth relative to adults (Jaureguizar et al., 2021). This is concerning given that adolescents are a high-risk demographic for spreading the virus (Bhatt et al., 2022). However, other research has found high rates of public health compliance among youth early in the pandemic (Dunn et al., 2021; Oosterhoff et al., 2020). Our results align with and extend this latter research, as youth reported fairly high levels of adherence to all public health measures in each cohort. Specifically, in the summer and winter cohorts, 49.0% and 42.0% of youth respectively minimized social interactions, 51% and 43% of youth respectively socially distanced, and 40% and 92% of youth respectively wore a mask in public. Moreover, the most common motivations for compliance were social responsibility and not wanting to get sick. The high rates of compliance are particularly important to highlight in the context of the numerous life disruptions that youth experienced throughout the pandemic, including social isolation (Ellis et al., 2020) and mental health concerns (Craig et al., 2022; Racine et al., 2021).

### Adherence to Public Health Measures

Although youth engaged in similar levels of social interaction in each cohort, representing the first two waves of the pandemic (C1 and C2, respectively), social distancing and mask wearing differed earlier relative to later in the pandemic. Relative to youth in C1, adolescents in C2 reported more mask wearing in public, but less social distancing. At the time of the C2 survey, public health measures had been ongoing for more than six months, pandemic fatigue may have set in, and rules about gathering with others had been in flux in many provinces. Our results are consistent with other longitudinal research finding that adherence to social distancing waned from the first to second wave of the pandemic (Aksoy, 2022; Franzen & Wöhner, 2021). Thus, it is not surprising that adolescents reported less social distancing compared to earlier in the pandemic. At the same time, mask wearing dramatically increased from C1 to C2, with only 40% of youth endorsing wearing a mask in public in C1 and 94% in C2. This jump may be partially explained by the mixed messaging from the WHO about the importance and efficacy of face masks early in the pandemic (Lewis, 2022; Tso & Cowling, 2020). The conflicting messages may have made it difficult for adolescents to understand the rationale for mask wearing, and as such, resulted in less adherence to this measure in C1 than C2. In addition, most provinces in Canada had mandated face masks in indoor public spaces by the time of the C2 survey, which likely bolstered adherence. Collectively, our results suggest that youth were in closer social proximity to one another as the pandemic progressed but used more masks while doing so.

The change in mask wearing may help us to understand the likelihood of youth continuing to comply with public health recommendations in the future. As provinces and countries remove mask mandates and rely on youth complying with recommendations out of their own volition, our results suggest that we may expect mask wearing to decrease substantially. This is a concern as people continue to contract COVID-19, putting those at risk in danger of contracting the virus.

### Motivations for Adherence to Public Health Measures

Understanding why adolescents engage or do not engage in public health measures can inform strategies to encourage compliance in future outbreaks and pandemics. In both cohorts, the most common motivations for engaging in public health measures were social responsibility and not wanting to get sick. This is consistent with Oosterhoff et al.’s (2020) finding that adolescents were highly motivated by social responsibility. However, governmental sanctions and parental enforcement were less commonly endorsed in our sample than in Oosterhoff et al.’s (2020) sample. This finding was consistent for youth who lived at home with their parents and those who lived in other locations (e.g., foster or kinship care). In our sample, being motivated by social responsibility was significantly associated with minimizing social interactions, social distancing, and mask wearing in C1, and with social distancing and mask wearing in C2. Furthermore, concern for population health was associated with minimizing social interactions, social distancing, and mask wearing in C1, and minimizing social interactions and social distancing in C2. Finally, not wanting to get sick was significantly associated with minimizing social interactions and mask wearing in both cohorts. Overall, these findings are consistent with the work of Patte and colleagues (2022), which found that youth were supportive of mask mandates if they perceived COVID-19 to be a risk. It is also in line with research that found low moral norms (i.e., lack of social responsibility) to be associated with lower public health compliance (Nivette et al., 2021). These findings add to the growing literature on public health adherence that underscores the salience of promoting social responsibility as a key factor in gaining compliance, particularly in youth. Moreover, these findings extend past work by demonstrating that motivations for adherence may differ depending on the specific public health measure. In particular, motivations for mask wearing seem to be more strongly driven by a desire not to get sick compared to social distancing.

One novel finding of this study is a shift in motivations in C1 compared to C2, particularly with mask wearing. Both parental enforcement and being concerned with population health were associated with greater adherence to mask wearing in C1 but not C2. It is possible that as pandemic fatigue set in, both parents and youth became less focused on protecting the broader society. Work published early in the pandemic found that messaging that induced empathy for people most vulnerable to the virus (e.g., the elderly or those with medical comorbidities) promoted greater motivation to adhere to social distancing and mask wearing measures (Pfattheicher et al., 2020). However, this messaging waned over time, which may have impacted adolescents’ motivations and parental enforcement. Alternatively, it could be that mask mandates in public spaces and schools became a driving factor and our item “*Province is in lockdown*” did not cover mask mandates. Echoing the ideas of Pfattheicher et al. (2020), as society moves forward in response to both COVID-19 and future pandemics, public health officials may want to integrate both informational content and emotional content in messaging related to COVID-19 measures to bolster adolescents’ concern and passion for broader society.

It is also important to examine predictors of nonadherence to public health measures. Notably, concern with maintaining social ties was negatively associated with both minimizing social interactions and social distancing in C1. This is not surprising given the importance of peer relationships in adolescence (Waldrip et al., 2008), and the fact that public health measures can significantly disrupt these relationships. To strengthen public health compliance in future outbreaks or pandemics, it may be important for adolescents to have alternative avenues for socializing with peers, such as structured ways to meet with friends online.

### Limitations

Several study limitations need to be acknowledged. First, our study relied on self-report data. Due to social desirability bias, adolescents may have over-reported their compliance with public health measures (Daoust et al., 2021). Nonetheless, it is critical to ask youth themselves about their motivations for and adherence to public health measures, as this potentially provides a more realistic and clear understanding of what youth think and feel about public health compared to external reporters (e.g., parents and teachers). Second, this study used a convenience sample, which may have attracted participants who were prone to engage in public health measures. Third, although we had two cohorts of data, our study did not track the same participants over time. This prevented investigation of within-person changes in compliance and motivations over time. Fourth, both cohorts were heavily skewed in both girl gender and White race/ethnicity, although representative of the Canadian population (Statistics Canada, 2017). Consequently, our results may not generalize to other populations. Fifth, due to concerns about participant fatigue, we relied on a single item to assess the important construct of concern around social relationships. Future research would benefit from multiple indicators of this construct. Finally, there was an administration error in the mask wearing variable that necessitated the need to collapse responses. This led to similar results with one difference; being motivated by social judgment had a small, negative association to mask wearing when the responses were not collapsed. However, due to the number of associations being examined, we selected stricter alpha values (*p* < .01), and thus, this association would not have been interpreted.

### Conclusions

The current study sheds light on adolescents’ adherence to and motivations for complying with public health measures at two different points of the COVID-19 pandemic (i.e., Summer 2020 and Winter 2020/2021) in Canada. Relatively high rates of adherence to public health measures were endorsed in each cohort, although youth engaged in more mask wearing but less social distancing as the pandemic progressed. Social responsibility and not wanting to get sick were the most common motivations for adhering to public health guidelines, and consistently predicted compliance with most guidelines throughout the pandemic. Finally, youth shifted away from adhering to some public health measures (i.e., mask wearing) due to concern with population health later in the pandemic. These results underscore the importance of appealing to youth’s sense of social responsibility, particularly to their families, in targeted campaigns to encourage compliance with public health measures among this population.

## Supplemental Material

Supplemental Material - Adherence to and Motivations for Complying With Public Health Measures Among Adolescents During the Coronavirus Disease (COVID-19) Pandemic in Canada Supplemental Material for Adherence to and Motivations for Complying With Public Health Measures Among Adolescents During the Coronavirus Disease (COVID-19) Pandemic in Canada by Stephanie G. Craig, Christina L. Robillard, Megan E. Ames, Samantha Feldman and Debra J. Pepler in Psychological Reports

## Data Availability

Data is available in aggregate form upon reasonable request.

## Notes

1. We also ran an independent sample *t* test where the response options of “a lot” and “a great deal” for mask wearing were not collapsed in C2. The results did not differ in interpretation to those presented in-text, *t* [1324.05] = 16.53, *p* < .001.

2. We also ran a path analysis model where response options of “*A lot*” and “*A great deal*” were not collapsed for the mask wearing variable in C2. The results were similar in interpretation to what is presented in text, but differed for being motivated by social judgment. In C2, mask wearing was positively associated with being in lockdown (*β* = .09, *SE* = .03, *p* = .007), social responsibility (*β* = .23, *SE* = .05, *p* < .001), not wanting to get sick (*β* = .17, *SE* = .04, *p* < .001), having nothing else going on (*β* = .07, *SE* = .03, *p* = .032), concern with family health (*β* = .09, *SE* = .04, *p* = .032), and concern with population health (*β* = .10, *SE* = .04, *p* = .015). It was negatively associated with being socially judged (*β* = −.08, *SE* = .04, *p* = .033).
