# Supplementary material for: Adherence to and Motivations for Complying With Public Health Measures Among Adolescents During the Coronavirus Disease (COVID-19) Pandemic in Canada
Source: Psychol Rep. 2023 Sep 21;128(5):3192–211. doi: 10.1177/00332941231201355 (PMC12394772; doi:10.1177/00332941231201355)
Supplement: Supplemental Material - Adherence to and Motivations for Complying With Public Health Measures Among Adolescents During the Coronavirus Disease (COVID-19) Pandemic in Canada [file sj-pdf-1-prx-10.1177_00332941231201355.pdf]

Supplemental Table 1. *Correlations Between Study Variables*

	1	2	3	4	5	6	7	8	9	10	11	12	13	14	15
1. Minimizing social interactions	-	.33**	.11**	.10*	-.02	.03	.05	.10**	.06	.17**	.09	-.03	.06	.05	-.05
2. Social distancing	.36**	-	.24**	.02	.03	.05	.14**	.11**	.03	.07	-.03	-.001	.17**	.13**	.08*
3. Mask wearing	.20**	.28**	-	.09*	.01	.01	.28**	.20**	.02	-.01	.04	.05	.17**	.15**	.08*
4. My province is on lock down	.08**	.07*	.07	-	.11**	.15**	.02	.15**	.10**	.18**	.12**	.07	-.002	.05	.11**
5. My parents are making me	.04	.01	.12**	.23**	-	.29**	.02	.19**	.16**	.11**	.22**	.10*	.07	-.03	.08*
6. My friends told me I should	-.01	.03	.07	.07*	.16**	-	.10**	.14**	.26**	.14**	.23**	.19**	.02	.04	.08
7. It is socially responsible	.18**	.21**	.25**	.17**	.10**	.09*	-	.30**	.05	.11**	.13**	.13**	.13**	.21**	.03
8. I don’t want to get sick personally	.18**	.17**	.23**	.27**	.19**	.14**	.43**	-	.06	.23**	.15**	.20**	.12**	.12**	-.001
9. There is nothing going on anyway	.02	-.11**	-.05	.10**	.11**	.19**	-.03	.04	-	.15**	.25**	.13**	.01	-.002	.02
10. I prefer to stay at home	.16**	.07	-.06	.09*	.001	.07*	.07	.19**	.15**	-	.09*	.09*	.01	.11**	-.08*
11. I don’t want to be socially judged	.00	.02	.05	.14**	.13**	.15**	.16**	.15**	.20**	.22**	-	.15**	-.02	.01	.10*
12.	-.05	.05	.06	.08*	.03	.01	.05	.12*	.08*	-.02	.08*	-	.11*	.06	.10*

	1	2	3	4	5	6	7	8	9	10	11	12	13	14	15
Work/home responsibilities								*					*		
13. Family health stress	.02	.16**	.17**	.13**	.10**	.04	.20**	.25**	.00	.02	.04	.07*	-	.42**	.15**
14. Population health stress	.15**	.24**	.25**	.13**	.05	.06	.31**	.21**	-.06	.01	.03	.05	.44**	-	.26**
15. Maintaining social ties stress	-.08*	-.05	.050	.04	.14**	.09*	.07	.09*	.03	-.09*	.08*	-.02	.20**	.26**	-

Note. Correlations below the diagonal are from C1 and correlations above the diagonal are from C2. *p < .05; * p < .001.
